# Novel Application of an Imageless Robotic System in Revision Total Knee Arthroplasty: Unicompartmental Knee Arthroplasty Conversion, 1‐Stage Revision, and 2nd‐Stage Revision

**DOI:** 10.1002/rcs.70135

**Published:** 2026-01-09

**Authors:** Joshua Yeuk Shun Tran, Rex Wang‐Fung Mak, Kevin Ki‐Wai Ho, Jonathan Patrick Ng, Cham Kit Wong, Gloria Yan‐Ting Lam, Tsz Lung Choi, Michael Tim‐Yun Ong, Patrick Shu‐Hang Yung

**Affiliations:** ^1^ Chinese University of Hong Kong Hong Kong China; ^2^ Prince of Wales Hospital Hong Kong China; ^3^ CUHK Medical Centre Hong Kong China; ^4^ Alice Ho Miu Ling Nethersole Hospital Hong Kong China

**Keywords:** imageless robotic systems, intraoperative mapping, revision total knee arthroplasty, robotic surgery, unicompartmental knee arthroplasty

## Abstract

**Introduction:**

Revision total knee arthroplasty (TKA) presents significant challenges due to factors such as infection, component failure, and bone loss. The application of the CORI imageless robotic systems in revision TKA remains underexplored. This study evaluates the utility of the imageless robotic system in three complex revision scenarios: unicompartmental knee arthroplasty conversion, one‐stage revision, and second‐stage revision.

**Methods:**

A prospective case series of patients undergoing revision of TKA was conducted. Intraoperative surface mapping and soft tissue tension guided surgical planning. Standardised perioperative protocols and rehabilitation were applied.

**Results:**

All patients demonstrated improved outcomes, with Knee Society Scores ranging from 90 to 95 and functional scores from 50 to 80 within two to six months postoperatively. Enhanced alignment and gap balancing were consistently achieved.

**Discussion:**

The imageless system facilitated precise intraoperative assessment and standardised techniques in revision TKA, supporting its potential to improve clinical outcomes. Further studies are warranted to establish long‐term benefits.

## 1 Introduction

Revision total knee arthroplasty (TKA) is a common procedure with various causes including infection, mechanical complications, and dislocation [1]. The approach to management and treatment of revision total knee arthroplasty varies according to the failure mechanism with the focus on minimising subsequent complications [2].

Another cause for revision TKA is unicompartmental knee arthroplasty (UKA) conversion. It is common for UKA knees to fail and thus require conversion to total knee arthroplasty (TKA) due to causes such as disease progression, component loosening or sinking, and unexplained pain [3, 4, 5]. Challenges of UKA conversion include varied UKA implants and alignments, intra‐articular scarring, implant and cement removal, and loss of bony landmarks post‐UKA [6, 7]. Despite the frequent need for conversion, there is yet to be a widely accepted consensus on the surgical techniques [8].

Imageless robotic systems may provide a platform for standardised techniques as they create an accurate intraoperative virtual 3D model via surface mapping and are able to obtain the operative soft tissue tension by applying varus and valgus stress through the full range of motion of the knee [9]. Furthermore, the imageless system does not rely on preoperative imaging to plan and execute accurate bone resections. This allows the surgeon to plan and execute the component position and soft tissue tension precisely and accurately according to the surgeon's preference.

Utilisation of imageless robotic surgical systems has shown significant improvements in both UKA [10] and TKA [11]. However, the evidence to support the use of imageless robotic systems in revision TKA remains scarce and inconclusive. Here we report on a novel use of an imageless robotic surgical system (CORI Surgical System, Smith&Nephew, London, UK) in revision knee arthroplasty with a case series: UKA conversion to TKA, 1‐stage revision TKA, and 2nd‐stage revision TKA.

## 2 Materials and Methods

This is a case series of prospectively collected patients who underwent revision knee arthroplasty with usage of an imageless robotic surgical system. The revision knee arthroplasties were conducted in a tertiary centre by the same team of experienced specialist orthopaedic surgeons from the arthroplasty division. Ethical approval was obtained from the Institutional Ethics Review Committee of the Joint CUHK‐NTEC Clinical Research Ethics Committee. Written informed consent by the patients is provided in the supplementary documents. All materials are original and reproduced with permission.

All TKA surgeries were performed using either mechanical or kinematic alignment based on the surgeon's preference. Identical wound closure techniques and a postoperative recovery protocol, including perioperative analgesic and antiemesis measures, were implemented as part of the adult joint reconstruction enhanced recovery after surgery protocol. A standardised physiotherapy rehabilitation protocol for adult joint reconstruction was followed and patients were discharged once their mobility allowed outpatient care.

## 3 Results

A total of three patients were recruited into the study (Table 1). The cases and specifics are detailed below.

**TABLE 1:** Peri‐operative characteristics.

	Case 1	Case 2	Case 3
Preoperative characteristics			
Height	155.4 cm	151.8 cm	159.1 cm
Weight	74.6 kg	66.3 kg	74.3 kg
Past medical history	Hyperlipidaemia, diabetes mellitus, hypertension	Hyperlipidaemia, hypertension, congestive heart failure	Hyperlipidaemia, diabetes mellitus, hypertension
Index surgery	Jan 2010	Apr 1998	Mar 2023
Complication	Mechanical knee pain	Mechanical knee pain	Prosthetic joint infection
Complication date	Apr 2016	Nov 2021	Mar 2024
Operation			
Revision surgery	Feb 2024	Sep 2024	Dec 2024
Surgery duration	120 min	190 min	206 min
Intra‐op blood loss	50 mL	150 mL	200 mL
Intra‐op range	0–130	0–130	0–120
Time to discharge	2 days	3 days	9 days

### 3.1 Case 1: UKA Conversion to TKA

**FIGURE 1:**
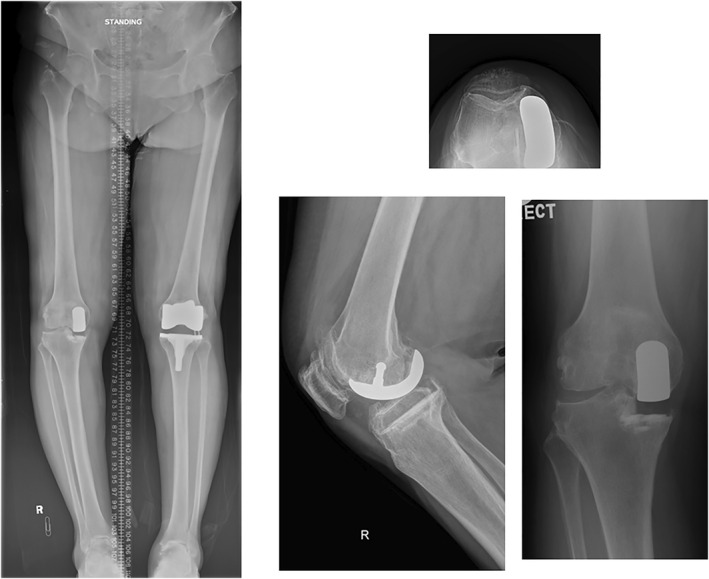
Case 1: Pre‐operative radiographs of Ms. F showing UKA in situ and osteoarthritis over the lateral and patellofemoral compartments.

Ms. F was a 74‐year‐old lady who had a history of right UKA done in 2010 for medial compartment OA. She complained of progressive right knee pain and deformity. Physical examination showed a medial parapatellar curvilinear incision. There was varus deformity of the knee with range of motion 10–120. Anterior drawer, varus and valgus stress tests were negative. There was focal tenderness over the medial and lateral joint lines, with Clarke's test positive. Standing radiographs showed a static tibiofemoral angle of 11° varus. The patellofemoral joint was preserved on the skyline view (Figure 1). In view of OA progression with increasing knee pain, the patient was consented for robotic‐assisted conversion TKR.

Skin preparation and draping were performed in standard fashion. A previous parapatellar curvilinear incision of the skin was incorporated with medial parapatellar arthrotomy. The implants were found to be well‐fixed with minimal wear. After performing arthrolysis and removal of osteophytes, tracker pins were inserted into the anteromedial aspect of the distal femur and distal tibia. Adequate distance from the joint line to tibial pins is paramount for insertion of the tibial stem, otherwise robotic assistance cannot proceed. The authors recommend 12–15 cm from joint line to allow for insertion of the shortest length tibial stem. For the femoral side, tracker pins may be inserted at the metaphysis perpendicular to the femoral shaft axis, and more proximally to allow adequate space for box reaming.

**FIGURE 2:**
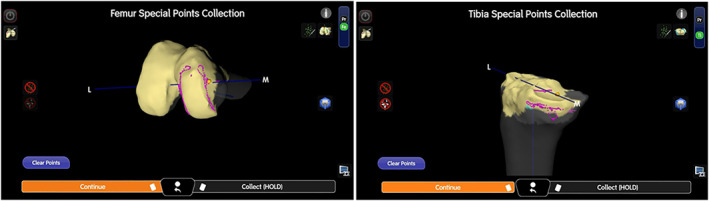
Case 1: Surface mapping was performed with implant in situ; subsequently the true bone surface was marked with ‘Special Points’ function of the system software after implant removal. The true bone surface is displayed as pink points on the screen.

While keeping the implant in situ, the hand‐held probe was used to perform surface mapping. Gap assessment is then performed using a Z‐shaped knee retractor or hand‐held electronic tensioning device (CORI Digital Tensioner, Smith & Nephew, London, UK). The knee is brought into full flexion and extension while applying varus and valgus stress to the joint. The UKA implants are then carefully removed with chisels and osteotomes. After removal of implants, the new bone surface of the femur as well as the tibia is then marked using the ‘Special Points’ function of the robotic system. The true bone surface can hence be visualised on the computer screen via pink dots (Figure 2). Gap assessment with the implant in situ allows for a more accurate gap assessment respecting the native knee tension, and the ‘Special Points’ function allows the surgeon to assess the true bone surface and to plan bone cuts, as well as any need for wedge augmentation.

**FIGURE 3:**
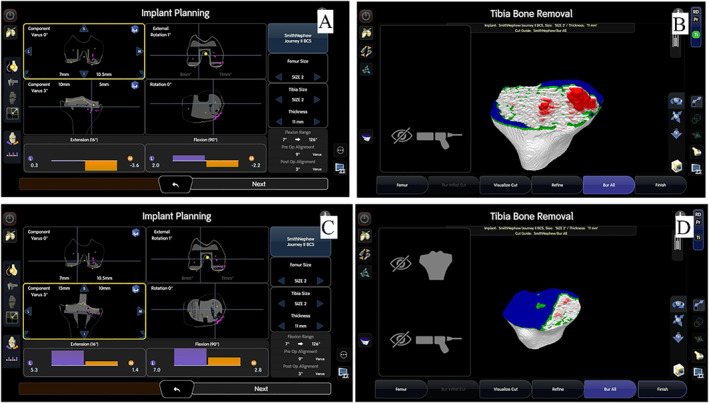
Case 1: Computer screenshot showing initial planning with desired tension (A); initial lateral plateau bone cut (B); tibial cut distalised 5 mm for medial augment (C); medial tibia bone cut for wedge augment (D).

Intra‐operative planning is then performed using the robotic system to ensure a well‐balanced medial and lateral gap according to surgeon preference. The authors routinely perform the tibia‐first technique in revision TKA. In UKA to TKA conversion, one would likely need to prepare for medial tibial wedge augmentation. Here the medial tibial bone defect can be easily visualised by the pink dots on the planning software, and the extent of defect may be evaluated by distalising the tibial bone cut to the level of the true bone surface. If the degree of bone loss is more than 2 mm, we suggest the use of wedge augment for more robust fixation, which would prevent early loosening and implant failure. This is achieved by first performing a tibial bone cut over the lateral tibial plateau using the original planned ligament tension and gaps. Subsequently, the bone cut for medial tibial augment is then performed by distalising the original tibial bone cut by multiples of 5 mm (i.e., to prepare for 5, 10 or 15 mm block augments) with the system software. During the medial augment bone cut, care must be taken not to remove too much bone laterally; the authors suggest marking the lateral edge of the augment with a diathermy beforehand. In our case, there was 4–5 mm bone loss over the medial tibial plateau; therefore, we opted for a 5 mm augment (Figure 3). Standard revision TKA can then be continued, starting with intramedullary reaming and determining tibial offset. We routinely utilise a short cemented stem with a metaphyseal cone for the tibia to ensure triple zonal fixation and decrease the risk of implant loosening. In UKA conversion, the femoral bone defect is usually minimal, and a standard bicruciate‐stabilised femoral component can be utilised in the absence of varus/valgus laxity. After confirming satisfactory flexion and extension gaps, tracker pins were removed and layered closure was performed in a standard fashion. No drain was inserted.

**FIGURE 4:**
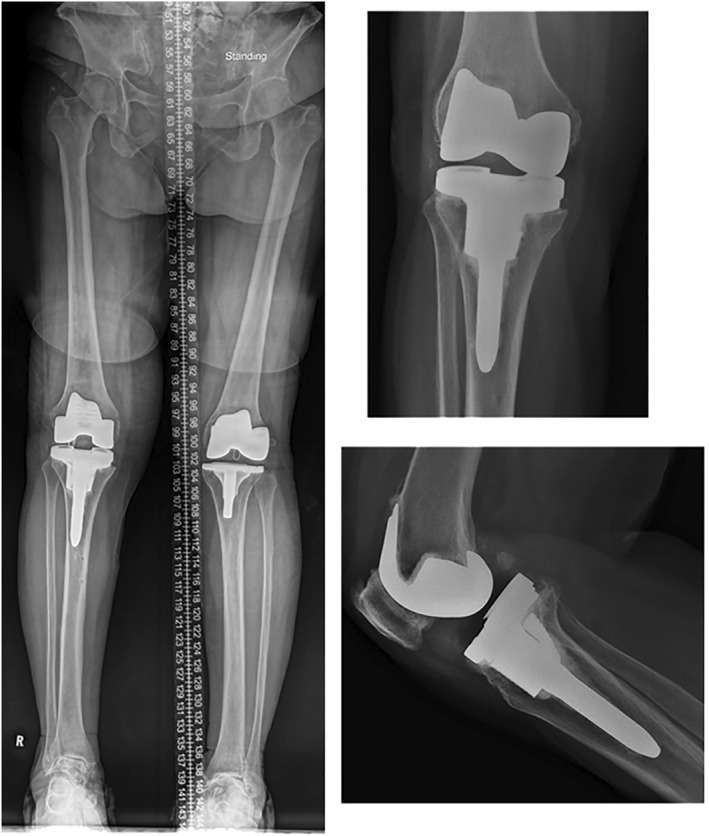
Case 1: Post‐operative radiographs of Ms. F.

Post‐operatively, Ms. F was allowed to weight‐bear as‐tolerated and she was discharged on day 3. She was last seen at 6 months post‐operatively, where she did not complain of any knee pain. She was able to walk without aids for more than 1 h. Her knee range of motion was 0–100 and her radiograph showed satisfactory alignment (Figure 4). Knee Society Score (KSS) was 95 and Knee Society Functional Score (KFS) was 80.

### 3.2 Case 2: 1‐Stage Revision TKR

**FIGURE 5:**
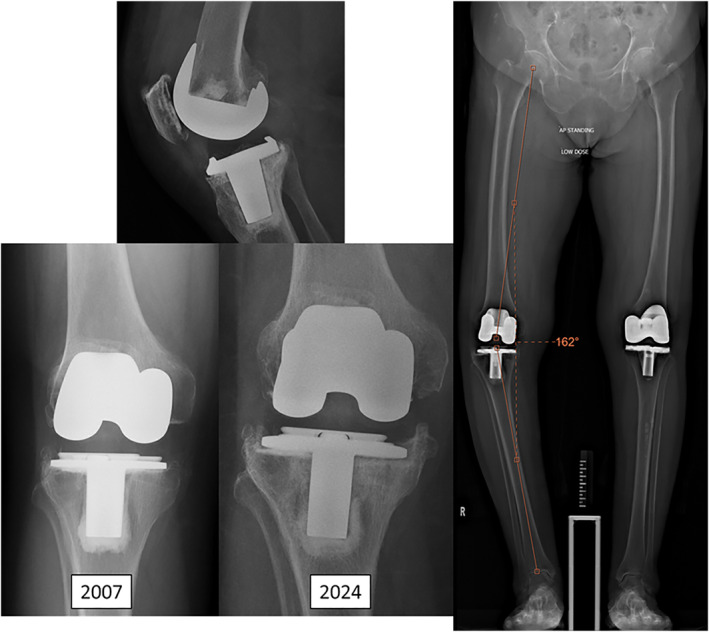
Case 2: Pre‐operative radiographs of Ms. T showing progressive tibial tray subsidence and a tibiofemoral angle of 18° varus.

Ms. T was an 84‐year‐old lady who had bilateral TKA performed in 1998 and 2007 for end‐stage knee osteoarthritis. Her right TKA, performed in 1998, was noted to have progressive tibial tray subsidence since 2021 (post‐op 23 years). She initially opted for observation as she was asymptomatic. She began to complain of increasing right knee pain in 2023 (post‐op 25 years). Radiography revealed further tibial tray subsidence and increasing varus deformity with a tibiofemoral angle of 18° varus (Figure 5). Knee range of motion was 0–110. The varus deformity was passively correctable to neutral, and the collateral ligaments were competent. She was consented for robotic‐assisted 1‐stage revision TKR.

**FIGURE 6:**
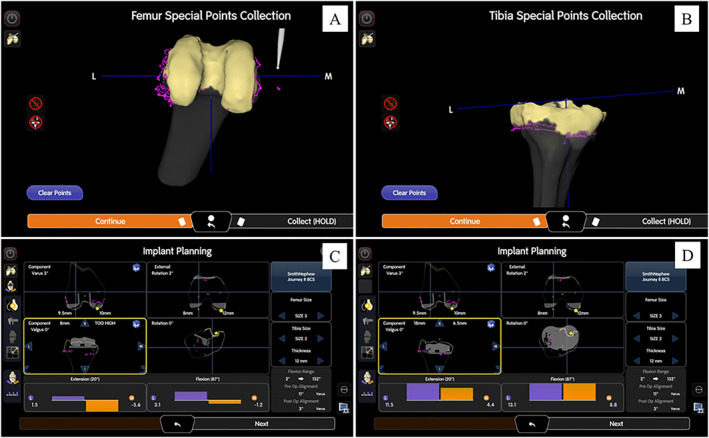
Case 2: Computer screenshot after surface mapping and removal of the implant. The true bone surface is displayed as pink dots on the screen (A, B). Implant planning can be done with the desired tension (C). The proximal tibial cut was then distalised 10 mm for medial and lateral augments (D).

**FIGURE 7:**
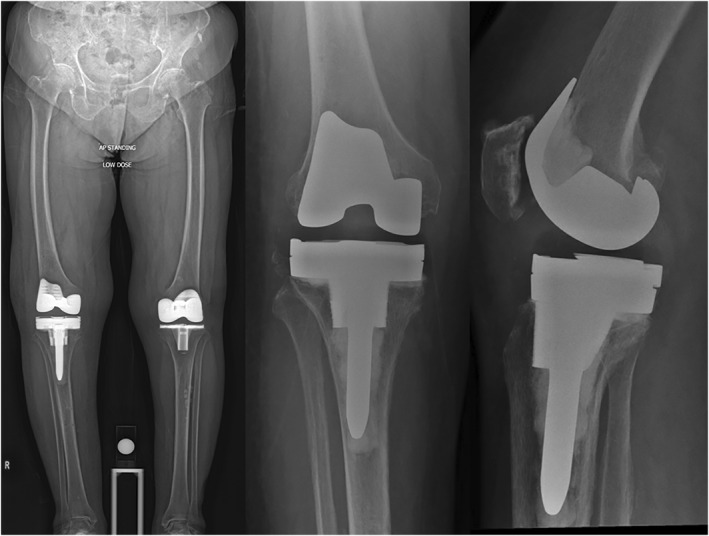
Case 2: Post‐operative images of Ms. T.

A previous midline incision and medial parapatellar approach was used. After intra‐articular soft tissue release, tracker pins were inserted as described in the technique above. Surface mapping and gap assessment were performed using the original prosthesis in situ. The prostheses can then be carefully extracted, and the true bone surfaces are marked with the hand‐held probe with the ‘Special Points’ function to determine the degree of bone loss. Initial implant planning is performed to achieve the desired soft tissue tension according to preference. Then, referencing the true bone surface via ‘Special Points’ function, the tibial cut should then be distalised to compensate for the bone loss and create a stable epiphyseal platform for augment placement (Figure 6). There was found to be uncontained bone loss over both tibial condyles (Anderson classification type 2B), and 10 mm medial and lateral augments were used for reconstruction. As before, short cemented stems and metaphyseal cones were used to ensure stable fixation. At the femoral side, there was minimal contained bone loss (Anderson type 1), and implant trials showed stable varus and valgus laxity. As such, a standard bicruciate stabilised femoral component and polyethelene liner were used (Figure 7). After standard closure, the patient was allowed to weight bear as tolerated on post‐operative day 1. She was discharged uneventfully on post‐operative day 3.

At 2 months weeks post‐operatively, her range of motion was 0–100 and she was able to ambulate with a stick for more than 1 h. KSS was 90 and KFS was 75.

### 3.3 Case 3: 2nd‐Stage Revision TKR

**FIGURE 8:**
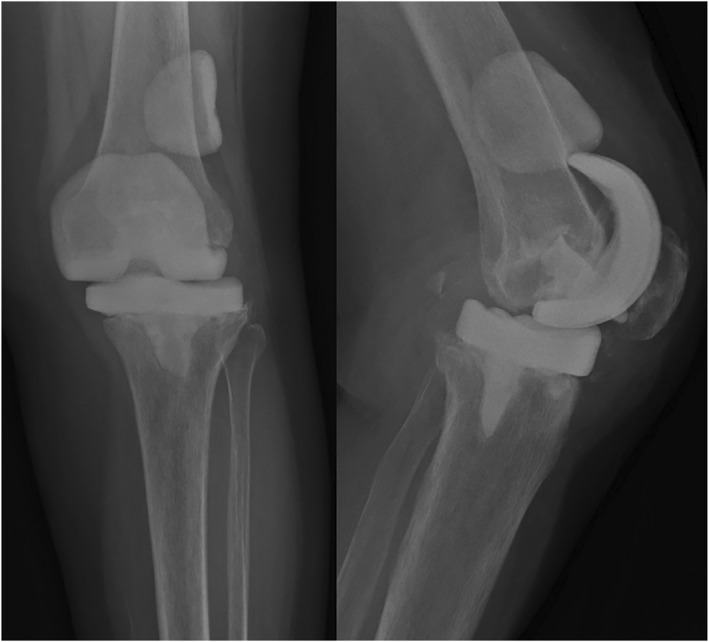
Case 3: Pre‐operative images of Ms. F.

Ms. F was a 67‐year‐old lady who had bilateral TKA performed in 2023 for end‐stage osteoarthritis of the knees. Her left TKA was complicated by Methicillin‐sensitive Staphylococcus Aureus prosthetic joint infection (PJI), which required removal of the implant and cement spacer, which was performed approximately 1 year after surgery (Figure 8). After completion of a full course of antibiotics, her blood inflammatory markers had normalised. Knee aspiration was performed, which did not show any residual microbial growth. She was subsequently consented for robotic‐assisted second‐stage TKA.

A previous incision and medial parapatellar approach was used. Intra‐articular release and insertion of tracker pins were performed. In this case, the femoral cement spacer was displaced, which would preclude accurate gap assessment throughout the entire knee range of motion. This is because the gap would be too large for instruments to accurately measure due to bone loss. We opted to place a trial metallic femoral component based on the size of the native femur, allowing us to perform the initial mapping and gap assessment. Cement spacers were removed and the true bone surface was recorded. There was mild subsidence of the tibial cement spacer resulting in symmetrical bone loss over the proximal tibia; 5 mm augments were used both medially and laterally. Metaphyseal cones and cemented stems were used. For the femur, there was minimal contained bone loss and a standard femoral component and insert were used.

**FIGURE 9:**
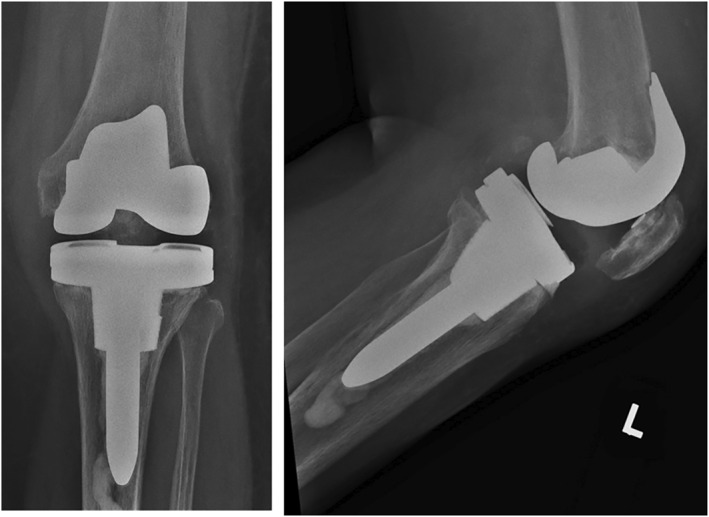
Case 3: Post‐operative images of Ms. F.

Ms. F was last seen at 2 months post‐operatively (Figure 9). She complained of mild knee pain and ambulated with a frame. Her knee ROM was 0–110 with KSS 90 and KFS was 50.

## 4 Discussion

To our knowledge, this is the first published case series detailing the use of the CORI surgical system for revision TKA. The operation is technically demanding, and the fixation of the implant and creation of a well‐balanced knee require more difficult techniques when compared to primary TKA [7]. This note shows the novel usage of surface mapping and Special Points collection to generate a precise and accurate virtual 3D model coupled with soft tissue tension assessment to achieve a well‐balanced revision TKA knee.

To explain in detail, surface mapping is first performed with the original implants left in situ and subsequently soft tissue tension of the native knee is determined. This is because the system software cannot proceed with soft tissue tension prior to surface mapping. Then, the implants are extracted, and the ‘Special Points’ function is utilised for accurate determination of the true bone surface. Using this function, pink‐coloured points can be visualised on top of the 3D model, especially during intra‐operative planning interface. This allows for accurate determination of bone cuts, especially if wedge augment is required for epiphyseal defects. There are a few disadvantages of the robotic system software. Firstly, the virtual 3D model is generated from previous CT scans collected from multiple patients. This means that the model is not a true representation of the actual anatomy, especially in patients with atypical anatomy. In primary TKA, simple surface mapping of the knee is sufficient to generate an accurate intraoperative 3D model [12]. However, simple surface mapping may produce inaccurate results for revision TKAs due to the distorted knee anatomy [13]. This can be alleviated using the ‘Special Points’ function of the robotic software to display the true bone surface. Secondly, the soft tissue tension must be performed with the original implant in situ, as it is difficult to collect tension data with implants removed, and robotic software is unable to properly display large gap values. This poses a risk of inaccurate gap determination if there is soft tissue release or injury during explantation. This may be avoided using meticulous surgical technique and careful explantation.

One ongoing concern during revision TKAs is bone loss associated with the surgery. Although bone cuts are necessary to correct malalignments and restore soft tissue tension, extreme amount of bone loss may result in subpar implant fixation and patient outcomes [14]. The integrated system from implant design to bone cutting of the CORI system enables exact and meticulous executions to maximise patient outcomes while ensuring adequate implant fixation.

Another important factor in achieving good revision TKA outcomes includes proper soft tissue balancing and tension [15]. However, good outcomes from manual soft tissue balancing through surgeon defined assessment may be difficult to achieve due to factors such as scarring from previous primary surgeries. With the addition of the imageless robotic system, real‐time gap assessment and balancing can be performed and correlated with the appropriate amount of bone cuts according to surgeon preference. As such, the system provides quantifiable real time data points during the surgery in addition to the surgeon‐defined assessment. This enables more standardised and reproducible results.

This technical note shows that imageless robotic surgical systems are an effective surgical option for revision TKAs that can make accurate and precise bone cuts and soft tissue balancing possible.

In conclusion, robotic‐assisted revision TKA is a safe, reproducible surgery allowing for a balanced and well‐aligned knee.

## Author Contributions

The authors confirm contribution to the paper as follows: Study conception and design: Joshua Yeuk Shun Tran, Rex Wang‐Fung Mak, Michael Tim‐Yun Ong. Data collection: Joshua Yeuk Shun Tran, Cham Kit Wong, Gloria Yan‐Ting Lam, Tsz Lung Choi, Rex Wang‐Fung Mak, Jonathan Patrick Ng, Kevin Ki‐Wai Ho, Michael Tim‐Yun Ong, Patrick Shu‐Hang Yung. Analysis and interpretation of results: Joshua Yeuk Shun Tran, Rex Wang‐Fung Mak. Manuscript preparation: Joshua Yeuk Shun Tran, Rex Wang‐Fung Mak. All authors reviewed the results and approved the final version of the manuscript.

## Ethics Statement

Ethical approval was obtained from the Institutional Ethics Review Committee of the Joint CUHK‐NTEC Clinical Research Ethics Committee.

## Consent

Written informed consent was obtained from the patients.

## Conflicts of Interest

The authors declare no conflicts of interest.

## Data Availability

Data available on request due to privacy/ethical restrictions.
